# REDUCING FINANCIAL TOXICITY IN THE HEALTH CARE SYSTEM USING COST ESTIMATION TOOLS FOR OLDER ADULTS

**DOI:** 10.1093/geroni/igad104.1862

**Published:** 2023-12-21

**Authors:** Robin Gelburd

**Affiliations:** FAIR Health, New York City, New York, United States

## Abstract

Financial toxicity— the financial, emotional and mental burden patients experience with medical costs that can lead to diminished access to care and reduced quality of life—is a growing issue among older adults, family caregivers and care partners. Research suggests that financial health literacy tools may reduce financial toxicity. With support from The John A. Hartford Foundation, FAIR Health undertook an 18-month initiative to advance shared decision making (SDM) among older adults (aged 65 and older) with serious health conditions, and their family caregivers/care partners, through the development and dissemination of decision aids that combine clinical and cost information with resources and educational content; these tools are offered for free to consumers on fairhealthconsumer.org and to healthcare providers on fairhealthprovider.org. This presentation will discuss salient findings from the initiative, as detailed in the brief, Advancing Shared Decision Making among Older Adults with Serious Health Conditions: Lessons from FAIR Health’s Grant-Funded Initiative. Feedback from older patients, caregivers/care partners and providers revealed the demand and need for financial health literacy tools that provide cost information and underscored key insights that included: (1) Healthcare costs, billing and lack of transparency in the healthcare system are sources of frustration; (2) Barriers to SDM include low health literacy and poor access to devices; and (3) SDM is viewed by many as a vehicle for patient empowerment. The session will offer a unique insight into how these findings can be used to usher in a new paradigm of healthcare and retool clinical practice and medical education.

